# Inertial-Measurement-Unit-Based Novel Human Activity Recognition Algorithm Using Conformer

**DOI:** 10.3390/s22103932

**Published:** 2022-05-23

**Authors:** Yeon-Wook Kim, Woo-Hyeong Cho, Kyu-Sung Kim, Sangmin Lee

**Affiliations:** 1 Department of Electrical and Computer Engineering, Inha University, Incheon 22212, Korea; kimywih1@naver.com (Y.-W.K.); wakeiy@naver.com (W.-H.C.); 2 Department of Otorhinolaryngology, Inha University Hospital, Incheon 22332, Korea; kyukim72@gmail.com; 3 Department of Smart Engineering Program in Biomedical Science & Engineering, Inha University, Incheon 22212, Korea

**Keywords:** inertial measurement unit, human activity recognition, data augmentation, conformer, transformer, convolutional neural network, multi-head self-attention

## Abstract

Inertial-measurement-unit (IMU)-based human activity recognition (HAR) studies have improved their performance owing to the latest classification model. In this study, the conformer, which is a state-of-the-art (SOTA) model in the field of speech recognition, is introduced in HAR to improve the performance of the transformer-based HAR model. The transformer model has a multi-head self-attention structure that can extract temporal dependency well, similar to the recurrent neural network (RNN) series while having higher computational efficiency than the RNN series. However, recent HAR studies have shown good performance by combining an RNN-series and convolutional neural network (CNN) model. Therefore, the performance of the transformer-based HAR study can be improved by adding a CNN layer that extracts local features well. The model that improved these points is the conformer-based-model model. To evaluate the proposed model, WISDM, UCI-HAR, and PAMAP2 datasets were used. A synthetic minority oversampling technique was used for the data augmentation algorithm to improve the dataset. From the experiment, the conformer-based HAR model showed better performance than baseline models: the transformer-based-model and the 1D-CNN HAR models. Moreover, the performance of the proposed algorithm was superior to that of algorithms proposed in recent similar studies which do not use RNN-series.

## 1. Introduction

Recently, as devices with built-in inertial measurement units (IMUs), such as smartwatches, fitness trackers, and smartphones, have become widespread, the interest in related research is growing. Studies on IMU-based human activity recognition (HAR) algorithms are a part of this research. IMU-based HAR technologies are expected to be utilized in digital healthcare; consequently, related studies have been steadily conducted over the past decades [1,2]. This includes a study on recognizing movements in the daily lives of people using smartphones [3], studies on recognizing sports movements [4,5], and studies on monitoring or assessing the motions of patients with Parkinson’s disease or brain disease [6,7].

Machine-learning techniques have been frequently used in IMU-based HAR studies [8,9,10]. In research using machine learning, it is important to extract handcrafted features. For this purpose, domain knowledge and signal processing theory are required. Recently, deep-neural-network (DNN)-based research has become increasingly popular. In deep learning models, the feature extraction process is performed automatically, and the resulting performance is excellent. Therefore, several DNN-based HAR studies have been conducted [2,11,12]. Furthermore, the recurrent neural network (RNN) series DNN model is structurally suitable for processing time-series data and is widely used. Long short-term memory (LSTM) and gated recurrent units (GRUs), which are types of RNN series models, feature a gate structure that can memorize the long-term state of the previous input and learn the sequential context of time-series data [13,14]. In addition, convolutional neural network (CNN) series models, which are widely used in the imaging field, are also frequently employed for time-series data. The CNN model can accurately extract spatial features from a spectrogram converted from a time series of data or multivariate time series. In addition, it offers the advantage of good computational efficiency, as compared with RNNs. Thus, many studies have attempted to develop LSTM and CNN ensemble models and achieved good performance [15,16].

Recently, transformer models that can extract temporal dependency information from sequential data have attracted attention. The transformer was first proposed in the field of speech recognition; it offers the advantage of better computational efficiency than RNN-series models and good extraction of long-term dependency information [17]. Thus, other data have also been introduced. Lim’s study [18] implemented a transformer-based multi-horizon forecasting model that efficiently learns long-term dependencies while being interpretable. The transformer’s efficient, global information capture and interpretable advantages were applied to the vision field and showed similar or superior performance to conventional CNN and RNN-based models [19,20,21]. Transformers have also been introduced in the field of HAR [22,23].

Similar to the transformer, the conformer was also proposed in the field of speech recognition. The conformer exhibits better performance than the current transformer and has, therefore, emerged as a state-of-the-art (SOTA) model in the field of speech recognition [24]. The conformer can extract temporal dependency, including positional embedding and multi-head self-attention (MHSA) structures, such as the transformer model. Unlike the transformer, however, the conformer incorporates a CNN structure to extract local features with slightly better performance [24,25,26]. Similarly, in recent HAR studies, the CNN model that extracts local features and the LSTM model that extracts temporal dependencies achieved better performance than the single-CNN model and the single-LSTM model. Therefore, when the conformer model is introduced, it is expected that performance will be improved compared to the existing transformer-based HAR model. Specifically, in this paper, we propose a conformer-based HAR algorithm. This study represents the first introduction of the conformer model in HAR research. To evaluate the performance of this model, the public WISDM, UCI-HAR, and PAMAP2 datasets were employed. Furthermore, a data augmentation technique was used to improve the dataset and the performance of the model. This data augmentation technique employed the synthetic minority oversampling technique (SMOTE) [27], which is widely adopted for time-series data augmentation.

## 2. Related Work

Deep learning algorithms have been widely used in recent IMU-based HAR research. Among them, RNN and CNN structures have been frequently used. Recently, the transformer model has been studied in many fields and has been recently introduced into IMU-based HAR research.

The RNN series model can extract temporal dependency of data and it is suitable for processing time-series data such as IMU-based HAR data. In the study of Okai et al. [28], a robust LSTM and GRU-based HAR model was proposed by approaching the data augmentation technique. The robustness of the LSTM and GRU HAR models was compared when sensor data were missing. In Zebin’s study [29], the LSTM with batch normalization applied to the HAR public dataset showed better accuracy than the LSTM model without batch normalization despite training with fewer epochs.

The CNN model extracts local features and shows good performance while being more efficient than the RNN-based model in time-series data. Kuang et al. [30] proposed the deep CNN model using dropout for HAR. The proposed CNN model showed better performance and training efficiency than the LSTM model. Teng et al. [31] proposed local loss-based CNN for HAR. The local loss-based model showed better performance than the global loss-based model at no extra cost. Sojeong et al. [32] proposed a 2D CNN model on multimodal HAR datasets. The approach showed better performance than various data mining techniques and 1D CNN model.

The CNN and RNN ensemble HAR models show good performance in HAR research by combining the local feature capability of CNN and the temporal dependency extraction capability of RNN series. Mekruksavanich [15] conducted a classification study using the smartwatch HAR public dataset in WISDM from the UCI repository; a CNN-LSTM model wherein the LSTM layer was located after the CNN layer was thus proposed. This model exhibited better performance than the CNN and LSTM models. Mukherjee [16] studied the HAR algorithm using a HAR public dataset. An ensemble model composed of three heads (CNN-LSTM net, CNN net, and encoded-net, which is an autoencoder including 1D-CNN) was thus constructed, and it exhibited good performance. Ordóñez [33] proposed the DeepConvLSTM HAR model, and this model showed better performance than the CNN-based HAR model in the public HAR datasets.

The transformer model is known to have good computational efficiency while extracting temporal dependency similar to the RNN model [17] and has been recently introduced into IMU-based HAR research. In Shavit’s study [22], the transformer encoder structure was used for the first time in HAR research. Input data were converted into a latent sequence embedding layer and then passed through a transformer encoder. The data were classified using a simple classification module. This previous model showed good performance for public HAR data. Furthermore, in the HAR study of Luptáková [23], the transformer model and data augmentation technique were applied, and the performance was significantly improved, as compared with that in previous machine-learning-based studies.

## 3. Materials and Methods

### 3.1. Dataset Description and Preprocessing

The performance of the proposed algorithm was evaluated using two public smartphone built-in IMU-based HAR datasets: WISDM and UCI-HAR.

Kwapisz et al. [34] developed the WISDM dataset. Motion data were collected for six movements (walking, jogging, ascending stairs, descending stairs, sitting, and standing) performed in daily life while carrying an Android smartphone (Nexus One, HTC Hero, and Motorola Backflip) in the front pant leg pocket. A smartphone app was provided for participants to label the motions themselves. Participants were asked to collect data while performing a specific set of motions for a specific time. Thirty-six participants participated, and three-axis linear accelerometer data were recorded at a sampling rate of 20 Hz. The execution time of participants’ motions were different from each other, and the data were imbalanced [34]. The total samples for each class were as follows: walking, 424,400 (38.64%); jogging, 342,177 (31.16%); upstairs: 122,869 (11.19%); downstairs, 100,427 (9.14%); sitting, 59,939 (5.46%); standing, 48,395 (4.41%). In this study, a sliding window size of 80 with a 50% overlap was applied.

The UCI-HAR dataset was developed by Anguita et al. [35]. Using a smartphone (Samsung Galaxy S II, Suwon, Korea) equipped at the waist, the motion data for six motions (walking, walking upstairs, walking downstairs, sitting, standing, and laying) performed in daily life were collected. Thirty participants aged 19–48 years participated in this experiment. Three-axis linear accelerometer and three-axis gyroscope motion data were recorded at a sampling rate of 50 Hz. The experiments were video-recorded for manual data labeling. The execution time of participants’ motions were slightly different from each other [35] and the data were not imbalanced. The total samples for each class were as follows: walking, 220,416 (16.72%); walking upstairs, 197,632 (14.99%); walking downstairs, 179,968 (13.65%); sitting, 227,456 (17.25%); standing, 243,968 (18.51%); laying, 248,832 (18.88%). The UCI-HAR dataset was segmented with each sample containing 128 timestamps with a 50% overlap. Therefore, the windowed data were used in this study.

To compare the model and performance of previous studies [36,37,38], data were divided in a manner consistent with previous studies. A random split method was used for both datasets; 70% of the data were used as training data with the remaining 30% used as testing data.

### 3.2. Data Augmentation

**Figure 1:**
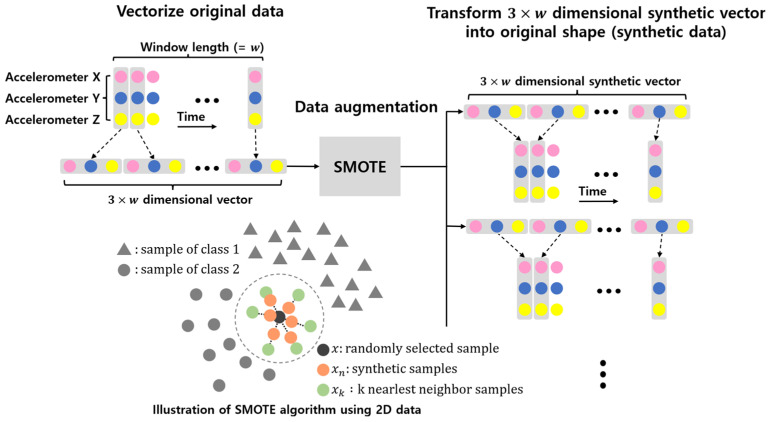
Processing windowed data before and after data augmentation and illustration of SMOTE algorithm using 2D data.

In classification problems, if there exists a significant difference in the amount of data for each class, the classification model biasedly trains the majority class, which may result in a lower classification accuracy [39]. One method to solve this problem is to equalize the amount of data in the majority and minority classes through data augmentation, which generates synthetic data similar to the original data [39,40,41]. In this study, synthetic data similar to the original data were generated using the oversampling technique, SMOTE [27]. Through data augmentation, the amount of data in each class was equalized; the amount of data was increased to improve the performance of the model. Steps 1–3 describe the data-augmentation process. The SMOTE algorithm augments windowed data because it has little effect on high-dimensional data for most trained classifiers [42]. The windowed data are 2D, and these 2D data are converted into a vector before applying the SMOTE algorithm. The vector is reshaped into original 2D data after data augmentation. Figure 1 illustrates the processing of windowed data before and after data augmentation and an illustration of SMOTE algorithm using 2D data.

1. The class set was $C_{s}$. The number of samples, *k*, with the closest Euclidean distance to a random sample, $x\;\left(x\;\epsilon \;C_{s}\right)$, which are windowed data, is $x_{k}\;(x_{k}\;\epsilon \;C_{s})$. $x_{k}$ was obtained using the k-nearest neighbor algorithm [27].
2. The number of n (n≤k) new samples between x and $x_{k}$ is $x_{n}$, and the rule for generating $x_{n}$ is expressed in Equation (1):
  (1) $$x_{n}=x+rand\left(0,1\right)\times \left|x-x_{k}\right|$$
3. Steps 1 and 2 are repeated such that the amount of class data in each class $(C_{0}\sim C_{5}$) becomes N.
4. K=10, $N_{WISDM}=12,000$, and $N_{UCI}=3500$ were applied using the augmentation process.

### 3.3. Proposed Model

#### 3.3.1. Conformer-Based HAR Model

**Figure 2:**
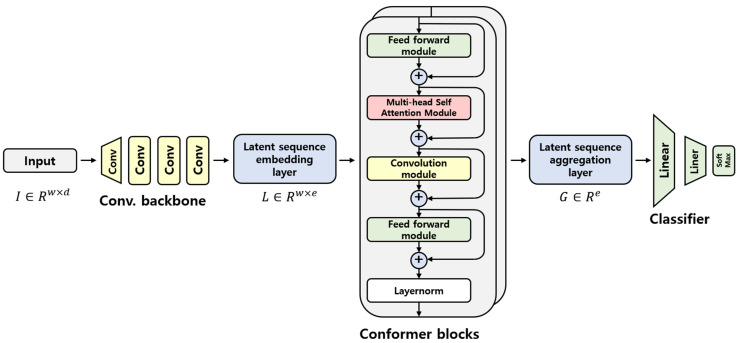
Proposed conformer-based HAR model.

The conformer-based HAR model proposed herein is depicted in Figure 2.

When the window size of the input data is w and the number of dimensions of the data is d, the input data are $I\in R^{w\times d}$. Then, the input data dimension is converted into e dimension by a convolutional backbone which is composed of four 1D-CNN layers and a GELU activation function. This converted layer is a latent sequence embedding layer, $L\in R^{w\times e}$. The conformer block receives information from the latent sequence embedding layer and extracts the features. The former block has a structure in which two feed-forward (FFN) modules sandwich the multi-head self-attention (MHSA) and CNN modules. In this study, the conformer block is composed of several layers; when the index of each conformer block is i and the output of the former block is $y_{i}$, the formula for the convolution block is as follows:

(2) $$\tilde{L_{i}}=L_{i}+\frac{1}{2}FFN\left(L_{i}\right)$$

(3) $$L_{i}^{\prime }=\tilde{L_{i}}+MHSA\left(\tilde{L_{i}}\right)$$

(4) $$L_{i}^{\prime \prime }=L_{i}^{\prime }+Conv\left(L_{i}^{\prime }\right)$$

(5) $$y_{i}=Layernorm\left(L_{i}^{\prime \prime }+\frac{1}{2}FFN\left(L_{i}^{\prime \prime }\right)\right)$$

After the output of the conformer block, the temporal dimension is aggregated into one dimension, and this layer is the latent sequence aggregation layer. This can be expressed as $G\in R^{e}$. Assuming that the output from n conformer blocks is $y_{n}$ and output of the latent sequence aggregation layer is Z, the formula is as follows:

(6) $$Z=y_{i}\left[:\right]\left[0\right]\left[:\right]\in R^{e}$$

After the aggregation layer, a simple classifier consisting of a fully connected layer is obtained. Normalization was first performed in the classifier. The input dimension was then reduced to $\frac{1}{4}$ with a linear layer, and the GELU activation function was applied. Subsequently, log softmax was applied to output the probability of belonging to the class.

#### 3.3.2. Training and Evaluation

An Adam optimizer was used to train the conformer-based-model. The learning rate was $10^{-4}$ and the weight decay was $10^{-4}$. The learning rate was varied according to the learning epochs using the optimizer scheduler, with a scheduler step size = 5 and γ = 0.5. The step size is the period of learning rate decay and γ is the multiplicative factor of learning rate decay. The batch size was determined experimentally, and it was set to 8 in WISDM and 4 in UCI-HAR; the epochs were set to 50. Configurations of experimental hardware are as follows: CPU—Intel XEON SCALEABLE GOLD 6230 × 2; RAM—DDR4 32G PC4-21300 × 8; GPU—NVIDIA GEFORCE RTX 3090 D6X 24GB × 4.

The evaluation metrics used were accuracy and the macro-average F1 score. The macro-average F1 score can determine whether a model can classify all classes well and it also evaluates how well the model handles imbalances [43]. Hence, the macro-average assigns every class the same importance value. The following is an explanation of the accuracy and macro-averaged F1 score. In a multiclass, the F1 score for each class was calculated in a one vs. rest manner. The predicted samples were classified into four categories.

1. Actual positives that are correctly predicted are called true positives (TP).
2. Actual positives that are wrongly predicted negatives are called false negatives (FN).
3. Actual negatives that are correctly predicted are called true negatives (TN).
4. Actual negatives that are wrongly predicted are called false positives (FP).

The accuracy is the ratio of the number of correct data points to the total amount of data predicted by the model:

(7) $$\mathrm{Accuracy}=\frac{\mathrm{TP}+\mathrm{TN}}{\mathrm{TP}+\mathrm{TN}+\mathrm{FP}+\mathrm{FN}}$$

The precision is the ratio of the correctly predicted positives to the total number of samples classified as positive.

(8) $$\mathrm{Precision}=\frac{\mathrm{TP}}{\mathrm{TP}+\mathrm{FP}}$$

The recall is the ratio of the correctly predicted positives to the actual number of positive samples.

(9) $$\mathrm{Recall}=\frac{\mathrm{TP}}{\mathrm{TP}+\mathrm{FN}}$$

The F1 score is the harmonic mean of recall and precision, and it is generally applied when datasets are unbalanced.

(10) $$F1\;\mathrm{score}=\frac{2\times \mathrm{Precision}\times \mathrm{Recall}\left(\mathrm{Precision}+\mathrm{Recall}\right)}{\mathrm{TP}+\mathrm{TN}+\mathrm{FP}+\mathrm{FN}}$$

The macro-averaged F1 score was defined as the mean of the class-wise F1 score, where i is the class index and N is the number of classes.

(11) $$\mathrm{Macro}\;\mathrm{averaged}\;F1\;\mathrm{score}=\frac{1}{N}\overset{N}{\underset{i=0}{\sum }}F1{\;\mathrm{score}}_{i}$$

## 4. Results and Discussion

### 4.1. Hyperparameter Parameter Optimization for the Model

The optimal hyperparameters of the proposed model were determined using the grid search method. Table 1 presents the optimal hyperparameters for WISDM and UCI-HAR. Dropout rates exist in the attention, feed-forward, and CNN layers of the conformer block [24].

**Figure 3:**
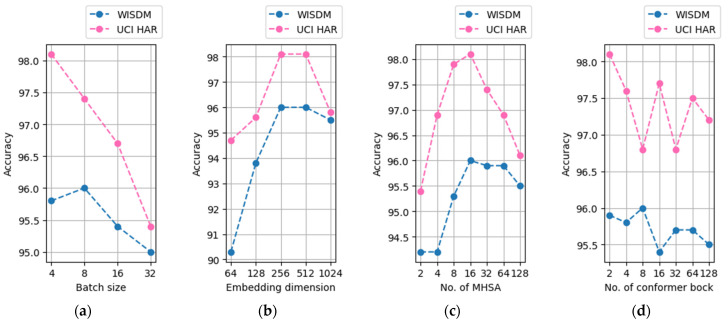
Optimal hyperparameters and batch size of the model; (**a**) accuracy according to batch size; (**b**) accuracy according to latent sequence embedding dimension; (**c**) accuracy according to the number of MHSA; and (**d**) accuracy according to the number of conformer block.

The major hyperparameters that had a significant influence on the performance of the model were the size of the latent sequence embedding dimension, the number of heads of MHSA, and the number of conformer blocks. In addition, the batch size influenced the performance. Figure 3 presents a graph expressing the accuracy before data augmentation when the latent sequence embedding dimension, number of MHSA heads, number of conformer blocks, and batch size were changed under optimal hyperparameter conditions.

When the batch size is small, the performance of the model increases. The UCI-HAR data afforded the best performance when the batch size was four, whereas for the WISDM data, when the batch size was more or less than eight, the performance decreased. Therefore, the optimal batch size was deemed to be eight. When latent sequence embedding dimensions are less than 256, the performance of the model increases as latent sequence embedding dimensions increase. When latent sequence embedding dimensions are over 256, the performance of the model is not increased. Therefore, the optimal latent sequence embedding dimensions were 256. Concerning the number of MHSA heads, the performance of the model tends to decrease to over or under 16 for both the UCI-HAR and WISDM data, and it was confirmed that 16 is optimal. Furthermore, the smaller the number of conformer blocks, the better the performance. The UCI-HAR data showed the best performance when the number of blocks was two. The optimal number of blocks for the WISDM data was eight. Overall, the performance tends to increase when the capacity of the conformer block and batch size is small; the performance also increases with the latent sequence embedding dimension, provided it is less than 256.

### 4.2. Evaluation of Proposed Algorithm

#### 4.2.1. Effect of Data Augmentation and Comparison of Proposed Model with Baseline Model

The deep learning model can be bias-trained on the majority class on an imbalanced dataset, which causes a performance decrease. To alleviate this phenomenon and improve the performance of the model, this study improved the WISDM and UCI-HAR data using data augmentation techniques.

The percentages of windowed data for each class in the WISDM dataset were as follows: walking, 9.17%; jogging, 32.17%; upstairs, 5.46%; downstairs, 4.39%; sitting, 11.17%; and standing, 38.64%. Thus, these were imbalanced data. The data augmentation algorithm, SMOTE, was adopted such that all the classes had the same amount of data as that of “walking,” which is a major class. The percentage of sliding windowed data for each class in the UCI-HAR dataset was as follows: walking, 16.72%; walking upstairs, 14.99%; walking downstairs, 13.65%; sitting, 17.25%; standing, 18.51%; and laying, 18.88%. Similar to the WISDM dataset, the SMOTE algorithm was used to equal the amount of UCI-HAR data for each class. To improve the performance of the model, the total amount of data was then doubled. Table 2 shows the performance of the experimental model before and after data augmentation.

The WISDM data indicated that the macro-averaged F1 score before augmentation was lower than accuracy. This was common for all the models. This phenomenon, however, was alleviated after data augmentation, implying that the data imbalance problem was resolved. After data augmentation, the conformer-based-model was improved for both datasets. On the WISDM dataset, the accuracy improved by 2.2%, and the F1 score improved by 3.5%. On the UCI-HAR dataset, the accuracy improved by 1.2%, and the F1 score improved by 1.1%. The degree of performance improvement was larger for the WISDM dataset than for the UCI-HAR dataset. This could be due to the alleviation of the data imbalance problem caused by data augmentation.

#### 4.2.2. Performance Comparison of the Proposed Algorithm with Baseline Models

To evaluate the performance of the proposed model, the transformer-based-model and the 1D-CNN model, proposed by Shavit [22], were tested together. For the direct comparison of the conformer and transformer structures, the remaining structures, except for the conformer and transformer structures, of the two models are made completely identical. Accordingly, the embedding dimension of Shavit’s transformer-based-model [22] was changed to 256, as in this study. Before and after data augmentation, the conformer-based-model showed superior accuracy compared to the transformer-based-model. To compare the training and test efficiencies of the models, the epoch and test times were also calculated. In the UCI-HAR dataset, the epoch times of the conformer-based-model were shorter than those of the transformer-based-model, that is, 16.7% and 15.7% shorter before augmentation and after augmentation, respectively, showing better training efficiency. Meanwhile, the test times of the conformer-based-model were longer, that is, 1.2% and 10.6% longer before augmentation and after augmentation, respectively. Furthermore, in WISDM data, epoch and test times of the conformer-based-model were much longer. However, the conformer-based-model’s capacity could be made similar to that of the transformer-based-model at a slight loss in accuracy. Therefore, the experiment was conducted by adjusting the number of conformer blocks from eight to two. Table 3 shows the performance of the conformer-based-model when the number of blocks is two.

When the number of conformer blocks is two, the epoch time of the model is 12.4% shorter before augmentation and 15.7% after augmentation than the transformer-based-model, showing better training efficiency. The test times of the conformer-based-model were 7.6% and 7.9% longer before augmentation and after augmentation, respectively, compared to the transformer-based-model.

As a result, the conformer-based-model had slightly lower test efficiency compared to the transformer-based-model but had better learning efficiency and accuracy.

#### 4.2.3. Comparison of Proposed Algorithm with Previous Studies

To evaluate performance, our proposed algorithm was compared with the algorithms used in previous studies [36,37,38]. These algorithms did not use the RNN series model but used similar algorithm evaluation techniques used in this study. In Ghate [36] and Khan’s study [38], the macro average F1 score was not used as a metric. Therefore, the algorithm in these studies and our algorithm were compared by considering accuracy. Table 4 lists the accuracies achieved in previous research and this study.

For the WISDM dataset, the performance of the proposed algorithm is 98.1%. This is consistent with the 98.2% accuracy achieved by the attention-based multi-head model, which showed the best performance among previous studies. The accuracy of the proposed model for the UCI-HAR dataset is 99.3%; this is superior to that of the DeepCNN-RF model, which afforded the best results among previous studies, by approximately 1.1%. Thus, the proposed algorithm showed good performance on both WISDM and UCI-HAR data, and its performance is superlative or even superior to that reported by previous HAR studies. The proposed algorithm not only has high performance but also has structural advantages due to the conformer. Because the conformer includes the MHSA structure, it can be more robust for long input lengths than CNN-based models [36,37,38], which cannot extract long-term dependencies. Because the MHSA structure of the conformer extracts long-term dependencies with better efficiency than the RNN-series model, it is more efficient than the CNN and RNN ensemble HAR models. Additionally, the conformer has the advantage of better extracting local features than the RNN-series single model. As such, the conformer-based-model can be said to be a model that always has an advantage no matter how it compares to any HAR model using CNN and RNN-series models.

#### 4.2.4. Verification of the Generality of the Proposed Model

An additional experiment was conducted to verify the generality of the model and whether the parameters in the WISDM data of the conformer-based-model work effectively in other IMU-based HAR data. The public HAR dataset used in this experiment is PAMAP2, which uses multi-channel IMU.

The PAMAP2 dataset was developed by Reiss et al. [44]. The three IMUs and temperature sensor were placed on the hand, chest, and ankle of each subject, and a heart rate sensor was used. Twelve daily activities (“Lying”, “Sitting”, “Standing”, “Walking”, “Running”, “Cycling”, “Nordic walking”, “Ascending stairs”, “Descending stairs”, “Vacuum cleaning”, “Ironing”, “Rope jumping”) data were collected. The accelerometer, gyroscope, magnetometer, and temperature data were collected with 100 Hz sampling rate and heart rate was collected with 9 Hz sampling rate. Nine participants aged 27–32 years participated in the experiment. Total collected data were around 10 h. A sliding window whose size was 100 and overlap of 50% was used for preprocessing data.

Table 5 shows the result of applying the conformer-based model for WISDM (conformer block = 2) to PAMAP2 and the result of applying the transformer-based-model (batch size = 8). Data augmentation doubled the total original data, such as UCI-HAR and WISDM. These data were generated so that there are 6500 data for each class.

As a result of the experiment in PAMAP2, it was confirmed that the accuracy and training efficiency of the conformer-based-model were superior to those of the transformer-based-model as in UCI-HAR and WISDM data.

To evaluate performance, our proposed algorithm was compared with the algorithms used in previous studies [45,46]. These algorithms did not use the RNN series model but used similar algorithm evaluation techniques used in this study. Table 6 shows accuracy of previous algorithms and proposed model in PAMAP2.

As a result of comparing the algorithm of this study with other studies, it was confirmed that the algorithm of this study showed better performance. As a result, it was confirmed that our proposed algorithm operates reliably on PAMAP2 data, which is another IMU-based HAR dataset.

## 5. Conclusions

In this study, a conformer, which is an SOTA model in the field of speech recognition, was introduced (for the first time) in HAR research. The structure of the conformer includes a CNN and an MHSA. The conformer showed better performance in the automatic speech recognition field than the transformer, which only included the MHSA. In deep-learning-based HAR research, because CNN and RNN series ensemble models have shown good performance, it is expected that the HAR model using the conformer-based-model would achieve better performance than that using the transformer-based-model. In this work, the WISDM and UCI-HAR datasets were used to evaluate the performance of the model, and the transformer-based-model and 1D-CNN models were additionally tested for a comparison with the proposed model. As expected, the conformer-based-model showed better performance than the transformer-based-model and 1D-CNN models. Additionally, data augmentation was performed to improve the model performance. The data augmentation algorithm used was the SMOTE algorithm, which has been widely used for time-series data augmentation. After data augmentation, the performance was improved for both the WISDM and UCI-HAR data. In particular, it was confirmed that the performance improvement was greater for the WISDM dataset, which comprises imbalanced data. In addition, the proposed algorithm was compared with previous HAR studies. These previous models were evaluated in a similar manner to in this study and did not use the RNN series as in this study. Based on the comparison, it was found that the performance of the proposed model was similar to or even better than that of previous models for both datasets. An additional experiment was conducted to verify the generality of the model and whether the parameters in the WISDM data of the conformer-based-model work effectively in other IMU-based HAR data. As shown in the UCI-HAR and WISDM datasets, the conformer-based-model performed better than the transformer-based-model, and showed better performance than the models of previous studies evaluated in a similar way without using an RNN structure.

This study mainly focuses on conformer-based HAR models. Although SMOTE-based data augmentation has shown the effect of improving the model’s performance, studies on data augmentation were a little lacking. It would be good to compare various data augmentation techniques and find a more effective technique for the HAR algorithm. Recently, data augmentation studies using deep-learning-based generative models such as the GAN and autoencoder have afforded good results [47,48,49]. As a follow-up study, it would be beneficial to investigate a deep-learning-based data augmentation algorithm suitable for HAR data. In addition, it is also possible to research to improve the efficiency and performance of conformer-based HAR model structures.

## Figures and Tables

**Table 1:** Optimal hyperparameters of the proposed model.

Parameter	WISDM	UCI-HAR
Latent sequence embedding dimension	256	256
Number of MHSA heads	16	16
Number of blocks	8	2
Feed-forward expansion factor	2	2
Convolution expansion factor	2	2
Dropout rates (%)	10	10

**Table 2:** Performance of proposed model before and after data augmentation.

Dataset	Metric	Conformer		Transformer		1D-CNN	
		Original	Augmented	Original	Augmented	Original	Augmented
WISDM	Accuracy (%)	96.0	98.1	95.5	97.9	85.7	89.1
	Macro F1 score (%)	94.6	98.1	94.2	97.9	80.3	88.9
	Epoch time (s)	115.1	285.3	100.5	243.5	22.8	53.6
	Test time (s)	157.0	392.9	62.9	148.4	35.0	79.0
UCI-HAR	Accuracy (%)	98.1	99.3	97.5	98.9	93.0	96.0
	Macro F1 score (%)	98.2	99.3	97.7	98.9	93.1	96.0
	Epoch time (s)	63.8	134.5	76.6	159.6	18.8	38.6
	Test time (s)	24.5	52.1	24.2	47.1	13.6	27.6

**Table 3:** Performance of the conformer-based-model when the number of blocks is 2.

Dataset	Metric	Conformer (No. of Conformer Block = 2)	
		Original	Augmented
WISDM	Accuracy (%)	95.9	98.1
	Macro F1 score (%)	94.5	98.1
	Epoch time (s)	87.99	205.3
	Test time (s)	67.7	160.1

**Table 4:** Comparison of previous algorithms and proposed model.

Algorithm	WISDM	UCI-HAR
	Accuracy (%)	Accuracy (%)
Proposed algorithm	98.1	99.3
DeepCNN-RF [36]	97.7	98.2
Fusion-Mdk-ResNet [37]	96.8	89.5
attention-based multi-head [38]	98.2	95.4

**Table 5:** Performance of proposed model before and after data augmentation in PAMAP2.

Dataset	Metric	Conformer for WISDM		Transformer	
		Original	Augmented	Original	Augmented
PAMAP2	Accuracy (%)	99.1	99.7	98.7	99.3
	Macro F1 score (%)	99.0	99.7	98.6	99.3
	Epoch time (s)	118.2	237.2	140.7	283.3
	Test time (s)	95.7	191.8	91.1	182.4

**Table 6:** Comparison of previous algorithms and proposed model in PAMAP2.

Algorithm	PAMAP2
	Accuracy (%)
Proposed algorithm	99.7
Linear grouped conv [45]	91.5
CondConv [46]	94.1

## Data Availability

The experiments have been carried out using sensor-based HAR datasets such as WISDM [34], UCI [35] and PAMAP2 [44] which are open for use in the research work.
